# Sodio urinario como factor pronóstico para mortalidad en pacientes con falla cardiaca aguda descompensada

**DOI:** 10.7705/biomedica.6920

**Published:** 2023-12-29

**Authors:** Jessica M. Londoño, Kelly J. Betancur, Lina Fonseca, Paula Fonseca, Eliana M. Cañas, Clara I. Saldarriaga

**Affiliations:** 1 Cardiología, Universidad Pontificia Bolivariana-Clínica Cardio VID, Medellín, Colombia Universidad Pontificia Bolivariana Universidad Pontificia Bolivariana Medellín Colombia; 2 Cardiología, Clínica Cardio VID, Medellín, Colombia Clínica Cardio VID Medellín Colombia; 3 Medicina Interna, Universidad Cooperativa de Colombia, Medellín, Colombia Universidad Cooperativa de Colombia Universidad Cooperativa de Colombia Medellín Colombia

**Keywords:** sodio, natriuresis, insuficiencia cardíaca, pronóstico, diuréticos, urgencias médicas, sodium, natriuresis, heart failure, prognosis, diuretics, emergencies

## Abstract

**Introducción.:**

En los pacientes con falla cardíaca, el sodio urinario se ha propuesto como marcador de gravedad y resistencia a los diuréticos, pero los resultados de los estudios reportados son heterogéneos.

**Objetivo.:**

Evaluar el sodio en orina ocasional como factor pronóstico de mortalidad en pacientes con falla cardiaca descompensada.

**Materiales y métodos.:**

Se realizó un análisis anidado de casos y controles de una cohorte prospectiva de falla cardíaca descompensada. El desenlace primario fue mortalidad a los 180 días. Se hizo un análisis bivariado para evaluar las variables que se asocian con la mortalidad. Se analizaron las diferencias de las variables clínicas entre los grupos con sodio urinario mayor o menor de 70 mEq/L.

**Resultados.:**

Se incluyeron 79 pacientes de los cuales 15 fallecieron a los 180 días. La edad promedio fue de 68,9 años (DE: ±13,8), 30 eran mujeres (38 %). Quince pacientes (18,9 %) tuvieron un sodio en orina inferior a 70 mEq/L. En el análisis bivariado se encontró una asociación significativa de la mortalidad con las hospitalizaciones, la presión arterial sistólica inferior a 90 mm Hg, el uso de inotrópicos y el sodio urinario inferior a 70 mEq/L. Los pacientes con sodio urinario bajo habían estado hospitalizados con mayor frecuencia en el último año, tenían menores valores de sodio sérico y presión arterial al ingreso.

**Conclusión.:**

Los pacientes con sodio urinario inferior a 70 mEq/L tienen características de mayor gravedad. En el análisis bivariado, el sodio urinario se asoció con mortalidad a los 180 días.

## Abstract (en)

**Introduction.:**

Urinary sodium has been proposed as a prognostic marker and indicator of the diuretic response in patients with heart failure. However, study results are heterogeneous.

**Objective.:**

To evaluate the spot urinary sodium level as a risk factor for mortality in patients with decompensated heart failure.

**Materials and methods.:**

We conducted a case-control study nested in a prospective cohort of patients with decompensated heart failure. The primary outcome was mortality at 180 days. The risk factors associated with mortality were evaluated through a bivariate analysis. Differences in clinical variables between groups with urinary sodium greater than or lesser than 70 mEq/L were analyzed.

**Results.:**

The study included 79 patients; 15 died at 180 days. Their mean age was 68.9 years (SD=± 13.8); 30 were women (38%), and 15 (18.9%) had urinary sodium <70 mEq/L. In the bivariate analysis, a significant association was found between mortality and past medical history of hospitalizations, SBP<90 mm Hg, the use of inotropes, and urinary sodium <70 mEq/L. Regarding clinical characteristics, patients with low urinary sodium level in the last year were hospitalized more frequently with hyponatremia and hypotension at admission.

**Conclusion.:**

Patients with urinary sodium <70 mEq/L had more severe signs. In a bivariate analysis, urinary sodium was associated with mortality at 180 days.

La falla cardiaca representa una carga significativa para los sistemas de salud y hace parte de las principales causas de morbimortalidad de la población. Se estima una prevalencia mundial en incremento que pasará del 2,4 % en el 2012 al 2,97 % para el año 2030 ^1^. Además, la tasa de admisiones hospitalarias en estos pacientes es muy alta, el 43 % reporta, al menos, cuatro hospitalizaciones en su seguimiento ^1^.

Colombia tiene una prevalencia aproximada de la enfermedad del 2,3 % y el 55 % de los pacientes que consulta el servicio de urgencias por una descompensación, tiene antecedentes de hospitalización previa por la misma causa. La media de la estancia hospitalaria es de tres a ocho días ^2^^-^^4^ y la mortalidad intrahospitalaria varía entre el 8,3 y el 9,2 % ^3^^,^^5^

El sodio urinario ha sido analizado en algunos estudios de falla cardíaca como un potencial marcador de desenlaces adversos, pero su forma de evaluación (orina ocasional versus recolección por un período determinado), el escenario clínico y los puntos de corte utilizados son muy variados ^6^^-^^10^.

Más allá de su utilidad en el pronóstico, en los últimos años, las principales sociedades de cardiología han recomendado utilizar el sodio urinario como un marcador de la respuesta al tratamiento diurético, basándose fundamentalmente en argumentos fisiopatológicos y en estudios pronósticos descritos. Incluso, proponen ajustar el tratamiento en caso de que el resultado esté por debajo del punto de corte establecido entre 50 y 70 mEq/L ^11^^-^^13^.

No se conocen estudios que validen la eficacia y seguridad del uso del sodio en orina en el servicio de urgencias para tomar decisiones terapéuticas. Sin embargo, debido a las recomendaciones descritas se ha incrementado la solicitud de este biomarcador. Particularmente, en la población colombiana no se ha estudiado el sodio urinario como marcador pronóstico.

Teniendo en cuenta los pocos estudios que utilizan el punto de corte sugerido por las sociedades científicas y la falta de datos en el país, el objetivo del presente estudio fue evaluar el sodio en orina ocasional como factor pronóstico independiente de mortalidad por cualquier causa a los 180 días, en pacientes con falla cardiaca agudamente descompensada que ingresaron por urgencias. La medición se realizó dos horas después de la primera dosis de diurético intravenoso con un punto de corte de 70 mEq/L.

## Materiales y métodos

Se realizó un análisis anidado de casos y controles en una cohorte prospectiva de pacientes con falla cardíaca descompensada en un centro de cuarto nivel de complejidad. La recolección de datos se realizó desde marzo hasta agosto del 2021.

### Criterios de inclusión

Se incluyeron personas mayores de 18 años con diagnóstico principal de falla cardíaca descompensada por su médico tratante al momento de su ingreso a urgencias. Se verificó que el paciente tuviera una condición de aparición rápida o empeoramiento de síntomas o signos de falla cardiaca, que implicara un cambio en el tratamiento crónico ^14^. En este estudio se consideró el uso de diurético intravenoso. Se incluyeron pacientes en todos los rangos de fracción de eyección (reducida, levemente reducida o preservada).

Se verificó la descripción, al menos, de dos de los signos o síntomas de congestión según las guías vigentes ^14^: disnea, ortopnea, disnea paroxística nocturna, edema periférico, bendopnea, aumento del pulso venoso yugular, presencia del tercer ruido cardíaco (S3) en la auscultación, reflujo hepatoyugular, crépitos pulmonares, derrame pleural, hepatomegalia, ascitis, signos congestivos en la radiografía de tórax o en la ecografía pulmonar, dilatación de la vena cava inferior, o elevación de péptidos natriuréticos (BNP>100 pg/ml o NT-proBNP>300 pg/ml).

Se tuvo en cuenta la disponibilidad del resultado del sodio urinario. Se incluyeron los pacientes a quienes se les tomó la muestra dos horas después del inicio del diruético intravenoso. El sodio urinario fue solicitado según el criterio del médico tratante.

### Criterios de exclusión

Se excluyeron los pacientes con una estancia hospitalaria menor de 24 horas, remitidos y hospitalizados en otra institución, que hubieran recibido terapia diurética intravenosa por más de 24 horas, con antecedentes de trasplante cardiaco, de haber participado previamente en el estudio, enfermedad renal crónica en diálisis y diagnóstico concomitante de otras enfermedades o condiciones agudas que condicionaran el pronóstico durante la hospitalización, como infección activa con sepsis grave, valvulopatía grave con criterio para corrección quirúrgica, infarto agudo del miocardio, ataque cerebrovascuolar agudo, trauma grave, choque hemorrágico, necesidad de cirugía mayor durante la hospitalización, intoxicación aguda, cirrosis descompensada o en estado de gestación.

Se hizo un seguimiento durante 180 días y se clasificaron como casos aquellos que cumplieron el desenlace primario de mortalidad por cualquier causa. Las variables demográficas y clínicas -incluyendo antecedentes y comorbilidades- se recopilaron en un formulario de Excel. Durante el ingreso y la hospitalizacion se registraron los signos vitales, la bioquímica básica, la dosis de diurético en las primeras 48 horas con el volumen urinario, y se definió un nivel bajo de sodio en orina menor o igual a 70 mEq/L ^11^.

El seguimiento se hizo con llamadas y revisión de las historias clínicas y la base de datos de la Administradora de los Recursos del Sistema General de Seguridad Social en Salud.

### Análisis estadístico

Las variables categóricas se expresaron en valores de frecuencias relativas y absolutas. Para las variables continuas se probó el supuesto de normalidad con la prueba de Kolmogórov-Smirnov y se expresaron en términos de media con desviación estándar (DE) o mediana con rango intercuartílico (RIC) según fuera pertinente.

Se realizó un análisis bivariado para evaluar las variables asociadas con la mortalidad. Se obtuvieron valores de razón de probabilidad *(odds ratio,* OR) con intervalos de confianza del 95 %. Se analizaron las diferencias entre las variables clínicas y de laboratorio con un punto de corte mayor o menor a 70 mEq/L. Se utilizó la prueba de X^2^ para comparar las variables cualitativas, la t de Student para las variables cuantitativas con distribución normal y la prueba de U de Mann-Whitney para las que no siguen una distribución normal. Se consideraron significativos aquellos resultados con valor de p inferior a 0,05.

Esta investigación se ajustó a las normas científicas para la investigacion y se considera sin riesgo, según la Resolución 8430 de 1993 del Ministerio de Salud de Colombia. El estudio fue aprobado por el Comité de Ética de la Escuela de Ciencias de la Salud y por Investigaciones de la Clínica Cardio VID. Todos los pacientes, como parte del protocolo de ingreso a la institución, firmaron un consentimiento informado que permite la recolección anónima de la información de su historia clínica con fines de investigación.

## Resultados

Un total de 307 pacientes cumplieron los criterios de ingreso a la cohorte de falla cardíaca aguda descompensada. Sin embargo, 98 pacientes tenían algún criterio de exclusión: hospitalización por menos de 24 horas (n=20); cirugía, infarto agudo de miocardio u otro diagnóstico principal diferente al de falla cardíaca (n=30); remitidos desde otra institución donde ya venían usando diurético intravenoso (n=5); antecedente de trasplante cardíaco (n=9) o reingreso al estudio (n=39).

De los pacientes ingresados, 79 individuos tenían disponibilidad de los niveles de sodio urinario a las dos horas del inicio del diurético, por lo que fueron incluidos en este subanálisis de casos y controles. De los 79 pacientes, 15 fallecieron en un período de 180 días de seguimiento y conformaron el grupo de casos. Como controles se analizaron los pacientes vivos en el mismo período de tiempo (n=63). Sólo hubo pérdida de seguimiento de un caso.

La media de la edad de los pacientes incluidos fue de 68,9 años (DE=13,8), el 38 % eran mujeres (n=30) y el 29 % de los individuos pertenecía a un programa especializado de falla cardíaca. La hipertensión, la fibrilación auricular, el tabaquismo y la diabetes fueron las comorbilidades más frecuentes (cuadro 1). La mediana de la fracción de eyección del ventrículo izquierdo (FEVI) fue del 30,5 %. Cincuenta y un pacientes (64,5 %) fueron clasificados con FEVI reducida, 10 pacientes (12,6 %), levemente reducida y en 18 pacientes (22,7 %), preservada. La etiología no isquémica fue la predominante (59,5 %) con la mayoría de los pacientes en clase funcional II (n=26; 32,9 %) y III (n=33; 41,8 %) (cuadro 1), según la clasificación de la *New York Heart Association.*

**Cuadro 1:** Características basales de la población general (N=79)

Característica	Pacientes
Sexo femenino (n, %)	30 (38)
Edad, media (DE)	68,9 (13,8)
Antecedentes	
Hipertensión arterial (n, %)	56 (70,9)
Diabetes mellitus (n, %)	26 (32,9)
Evento cerebrovascular (n, %)	6 (7,6)
Enfermedad pulmonar obstructiva crónica (n, %)	15 (19)
Enfermedad renal crónica (n, %)	17 (21,5)
Fibrilación auricular (n, %)	37 (46,8)
Tabaco (n, %)	34 (43)
Hospitalización en el último año (n, %)	34 (43)
FEVI, mediana (RIC) (n, %)	30,5 (23-45,5)
AHA D* (n, %)	12 (15,2)
Etiología	
Isquémica (n, %)	32 (40,1)
No isquémica (n, %)	47 (59,5)
Clase funcional NYHA	
I (n, %)	5 (6,3)
II (n, %)	26 (32,9)
III (n, %)	33 (41,8)
IV (n, %)	15 (19)
Dispositivo	
Resincronización cardiaca (n, %)	8 (10,1)
Desfibrilador (n, %)	7 (8,9)
Tratamiento	
IECA/ARA (n, %)	33 (41,8)
ARNI (n, %)	12 (15,2)
iSGLT2 (n, %)	14 (17,7)
ß-bloqueadores (n, %)	58 (73,4)
Antialdosterónicos (n, %)	38 (48,1)
Ivabradina (n, %)	3 (3,8)
Digoxina (n, %)	4 (5,1)
Furosemida (n, %)	58 (73,4)

DE: desviación estándar; FEVI: fracción de eyección del ventrículo izquierdo; RIC: rango intercuartílico; AHA: *American Heart Asociation;* NYHA: *New York Heart Association* IECA/ARA: inhibidores de la enzima convertidora de angiotensina / antagonistas del receptor de angiotensina II; ARNI: inhibidores de neprilisina; iSGLT2: inhibidores del cotransportador de sodio-glucosa 2

* AHA D: clasificación

En el cuadro 2 se muestran las características de los casos y los controles, junto con el análisis bivariado para determinar los factores de riesgo de mortalidad. Se encontró una asociación significativa entre la mortalidad y el antecedente de hospitalizaciones, la presión arterial sistólica menor de 90 mm Hg, el uso de inotrópicos y el sodio urinario menor de 70 mEq/L. No fue posible realizar un análisis multivariado por el tamaño de muestra.

**Cuadro 2:** Factores asociados con la mortalidad de pacientes con falla cardíaca descompensada a los 180 días

Característica	Vivos	Fallecidos	OR	P
	(n=63)	(n=15)		
	n %	n %		
Edad > 65 años	36 (57,1)	11 (23,4)	2,06 (0,59-7,18)	0,380
Sexo masculino	38 (60,3)	10 (66,7)	1,31 (0,40-4,30)	0,772
Antecedentes				
Hipertensión arterial	41 (65,1)	14 (93,3)	7,51 (0,92-60,9)	0,055
Diabetes mellitus	20 (31,7)	5 (33,3)	1,07 (0,32-3,56)	>0,999
Evento cerebrovascular	4 (6,3)	1 (6,7)	1,05 (0,10-10,1)	>0,999
Enfermedad renal crónica	12 (22,2)	3 (20)	0,87 (0,21-3,54)	>0,999
Tabaquismo	27 (42,9)	7 (46,7)	1,16 (0,37-3,61)	>0,999
EPOC	12 (19)	3 (20)	1,06 (0,25-4,36)	>0,999
Hospitalización previa	22 (34,9)	12 (80%)	7,45 (1,9-29,2)	0,003
FEVI < 50%	48 (77,4)	12 (20)	1,16 (0,28-4,72)	>0,999
NYHA III/IV	36 (57,1)	11 (73,3)	2,06 (0,59-7,18)	0,380
Etiología isquémica	24 (38,1)	7 (46,7)	1,42 (0,45-4,42)	0,569
PAS <90 mm Hg	1 (1,6)	4 (26,7)	22,54 (2,29-221,12)	0,004
PAD <60 mm Hg	3 (4,8)	3 (20)	5 (0,89-27,81)	0,081
Frecuencia cardíaca >100 Ipm	18 (28,6)	3 (20)	0,62 (0,15-2,48)	0,747
Hemoglobina < 12 mg/dl	13 (21)	5 (33,3)	1,88 (0,54-6,48)	0,323
Sodio sérico < 135 mg/dl	14 (22,2)	5 (33,3)	1,75 (0,51-5,96)	0,503
Sodio en orina < 70 mEq/L	8 (12,7)	6 (40)	4,58 (1,28-16,34)	0,023
Necesidad de inotrópicos	2 (3,2)	4 (26,7)	11,09 (1,80-68,09)	0,011
Tasa de filtración glomerular	32 (74,4)	11 (25,6)	2,664 (0,76-9,26)	0,153
<60 ml/min				

EPOC: enfermedad pulmonar obstructiva crónica; FEVI: fracción de eyección del ventrículo izquierdo; NYHA: *New York Heart Association;* PAS: presión arterial sistólica; PAD: presión arterial diastólica

Se compararon las variables clínicas y bioquímicas de los pacientes según el nivel de sodio en orina (cuadro 3). Quince pacientes (18,9 %) tuvieron un valor menor a 70 mEq/L a las dos horas del inicio del diurético intravenoso y solo cuatro de estos tuvieron un valor inferior a 50 mEq/L (5 %). Al observar los antecedentes, en general, los pacientes con sodio en orina bajo (con punto de corte de 70 mEq/L) tenían mayor edad, mayor carga de comorbilidades y mayor proporción de hospitalizaciones en el último año, pero solo esta última variable alcanzó la significancia estadística.

**Cuadro 3:** Comparación de las características basales de la población según el valor de sodio urinario menor o igual o superior a 70 mEq/L

Característica	Sodio urinario	Sodio urinario	p
	≤70 mEq/L (n=15)	>70 mEq/L (n=64)	
	n (%)	n (%)	
Sexo femenino (n, %)	4 (26,7)	26 (40,6)	0,386
Edad (años), media (DE)	74,2 (11,6)	67,7 (14,1)	0,127
Antecedentes			
Hipertensión arteria (n, %)	12 (80)	44 (68,8)	0,533
Diabetes mellitus (n, %)	10 (66,7)	21 (32,8)	>0,999
Evento cerebrovascular	2 (13,3)	4 (6,3)	0,319
Enfermedad pulmonar obstructiva crónica	4 (26,7)	11 (17,2)	0,467
Enfermedad renal crónica	6 (40)	11 (17,2)	0,079
Fibrilación auricular	7 (46,7)	30 (46,9)	>0,999
Tabaco	9 (60)	25 (39,1)	0,159
Hospitalización último año	10 (66,7)	24 (37,5)	0,048*
FEVI; mediana (RIC)	30 (20-50)	31 (23-45)	0,765
AHA D*	3 (20,0)	9 (14,1)	0,572
Etiología			0,771
Isquémica	7 (46,7)	25 (39,1)	
No isquémica	8 (53,3)	39 (60,9)	
Clase funcional NYHA			0,893
I	1 (6,7)	4 (6,3)	
II	6 (40)	20 (31, 3)	
III	5 (33,3)	28 (43,8)	
IV	3 (20)	12 (18,8)	
Dispositivo			0,420
Resincronización cardiaca	3 (20)	5 (7,8)	
Desfibrilador	1 (6,7)	6 (9,4)	
Tratamiento			
IECA/ARA	5 (33,3)	28 (43,7)	0,462
ARNI	3 (20,0)	9 (14,0)	0,689
iSGLT2	5 (33,3)	9 (14,0)	0,079
β-bloqueadores	11 (73,3)	47 (73,4)	>0,999
Antialdosterónicos	9 (60,0)	29 (45,3)	0,393
Ivabradina	2 (13,3)	1 (1,5)	0,091
Digoxina	1 (6,7)	3 (4,6)	0,577
Furosemida	12 (80)	46 (71,8)	0,747

DE: desviación estándar; FEVI: fracción de eyección del ventrículo izquierdo; RIC: rango intercuartílico; AHA: *American Heart Asociation;* NYHA: *New York Heart Association;* IECA/ARA: inhibidores de la enzima convertidora de angiotensina / antagonistas del receptor de angiotensina II; ARNI: inhibidores de neprilisina; iSGLT2: inhibidores del cotransportador de sodio-glucosa 2

* AHA D: clasificación

Entre las variables hemodinámicas y de laboratorio, los pacientes con sodio en orina menor de 70 mEq/L tenían valores de presión arterial sistólica y diastólica significativamente menores, así como valores bajos de sodio sérico. Se observó una tendencia de valores más altos de creatinina (cuadro 4).

**Cuadro 4:** Perfil hemodinámico, bioquímico y desenlaces de la población general y según el nivel de sodio urinario

Característica	Total	Sodio urinario	Sodio urinario	p
	N=79	≤70 mEq/L (n=15)	>70 mEq/L (n=64)	
PAS, mediana (RIC)	124 (112-143)	111 (97-122)	129 (115-145)	0,001
PAD, mediana (RIC)	78 (71-87)	71 (60-76)	79,5 (74-88)	0,010
Sodio sérico (mEq/L)	137 (135-140)	135 (132-137)	138 (138-140)	0,015
Potasio sérico (mEq/L)	4,29 (3,82-4,65)	4,30 (3,82-4,68)	4,29 (3,80-4,59)	0,841
Creatinina sérica (mg/dl)	1,18 (0,96-1,76)	1,5 (1,04-2,19)	1,15 (0,90-1,73)	0,081
Sodio urinario (mEq/L)	104 (83-122)	55 (45-63)	112 (98-125)	<0,001
Dosis de furosemida al ingreso (mg)	40 (40-80)	80 (40-80)	40 (40-80)	0,907
Volumen de diuresis a las 48 horas (ml)	1.965 (1.753-3.905)	2.980 (1.862-4.427)	2.965 (1.683-3.835)	0,797
Tratamiento hospitalario				
Inotrópicos	6 (7,6)	2 (13,3)	4 (6,3)	0,319
Vasopresores	1 (1,3)	0	1 (1,6)	>0,999
Muerte a los 180 días (n=78) *	15 (19,2)	6 (42,9)	9 (14,1)	0,023
Reingresos a 180 días (n=73) *	42 (56,0)	7 (46,6)	36 (56,25)	0,528

PAS: presión arterial sistólica; PAD: presión arterial diastólica

* Hubo 5 muertes intrahospitalarias y una pérdida de seguimiento

El desenlace principal de mortalidad a los 180 días fue mayor en los pacientes con sodio bajo (42,9 % versus 14,1 %; p=0,023). Las demás características fueron similares desde el punto de vista estadístico (cuadro 4).

## Discusión

En este estudio se encontró que los pacientes con diagnóstico de falla cardíaca aguda descompensada con congestión y sodio urinario bajo (<70 mEq/L) tienen mayor mortalidad. En el análisis bivariado, tanto el sodio urinario como la hipotensión y el requerimiento de inotrópicos durante la hospitalización se asociaron con un mayor riesgo de muerte a los 180 días.

La subpoblación de pacientes con sodio bajo en orina presenta diferencias significativas en otras características que representan mayor riesgo, características que, a su vez, hacen parte de la definición de falla cardíaca avanzada: hospitalizaciones recientes, presión arterial y sodio sérico bajos.

Este estudio definió el sodio urinario bajo como un resultado por debajo de 70 mEq/L en una muestra tomada dos horas luego del inicio del diurético intravenoso (n=15; 18,9 %). El punto de corte de sodio en orina ocasional sugerido por la Sociedad Europea de Cardiología para la toma de decisiones está entre menor de 50 y 70 mEq/L ^11^. Es llamativo que, en esta cohorte con empeoramiento de falla cardíaca, solo cuatro de los 15 pacientes con sodio urinario bajo, tuvieron un valor inferior a 50 mEq/L (5 %).

Los resultados de este estudio sugieren que un punto de corte inferior a 70 mEq/L podría llevar, más a menudo, a cambios en las conductas terapéuticas, dada su mayor frecuencia en la población con falla cardíaca descompensada, pero no existe ningún estudio que valide la eficacia y seguridad de establecer un límite específico de este biomarcador para tomar decisiones terapéuticas ^15^. Por otro lado, múltiples estudios han explorado el sodio en orina como factor pronóstico, pero la metodología utilizada para su medición varía considerablemente, muchos utilizan periodos de recolección continua de orina desde 6 hasta 72 horas ^15^^-^^17^. Un estudio encontró una correlación matemática entre el nivel de sodio en orina ocasional y el excretado en seis horas ^18^, pero esta equivalencia no ha sido reproducida, ni se utiliza rutinariamente.

Luego de la recomendación de la Sociedad Europea de Cardiología sobre un punto de corte específico en el paciente con descomposición aguda, se encontraron tres reportes que evalúan el sodio en muestra de orina ocasional como factor pronóstico luego del inicio del diurético intravenoso; a continuación, se establecen las semejanzas y diferencias con la cohorte del presente estudio.

Galluzo *et al.*^19^, en un subanálisis del estudio DRAIN que incluyó 80 pacientes, encontró un valor de sodio en orina menor de 50 mEq/L en el 35 % de la población. Sin embargo, estos pacientes tenían falla cardíaca avanzada con hiponatremia y se encontraban en la unidad de cuidados intensivos, lo que limita la utilidad de este punto de corte en esta subpoblación de la enfermedad. De la Espriella *et al.*^20^ encontraron una prevalencia de sodio urinario bajo con este mismo punto de corte en el 14,4 % de su cohorte, pero se trataba de pacientes seleccionados con falla cardíaca aguda y disfunción renal. No obstante, este resultado muestra una asociación entre la baja respuesta al diurético y la mortalidad a mediano plazo -mediana de seguimiento de 1,73 años- en esta subpoblación.

Por su parte, Luk *et al.* utilizaron un punto de corte de 60 mEq/L en una cohorte de 103 pacientes hospitalizados con diagnóstico de falla cardíaca descompensada. El 30 % de los pacientes tenía el sodio en orina por debajo del punto de corte, asociado también con mayor mortalidad y un mayor riesgo de requerir inotrópicos ^21^. Estos pacientes hacían parte de un programa especializado de falla cardíaca.

Honda *et al.* utilizaron el sodio en orina ocasional al ingreso y lo estudiaron como factor pronóstico, pero no tomaron la muestra con una relación temporal fija con el diurético ^22^. Dividieron su población en tres terciles de sodio, el más bajo tenía un punto de corte de 74 mEq/L y se caracterizó, al igual que la cohorte de este estudio, por una frecuencia mayor de antecedentes de hospitalización por falla cardíaca y niveles menores de depuración de creatinina, presión arterial sistólica y sodio sérico.

Así, los resultados de la cohorte del presente reporte concuerdan con aquellos que han utilizado una metodología y punto de corte similar para la medición del sodio urinario, y dan fuerza -en este contexto nacional- a su utilidad pronóstica. Además, la cohorte incluida proviene de una población menos seleccionada que las descritas previamente, lo cual es una fortaleza de este estudio, pues permite generalizar los resultados y da una visión amplia del comportamiento del sodio urinario en una cohorte de pacientes con falla cardíaca descompensada y congestión en todo el espectro de la enfermedad.

A pesar de la concordancia entre los estudios descritos y los hallazgos en la cohorte de este estudio respecto al valor pronóstico que tiene el sodio urinario en los pacientes con falla cardíaca descompensada, aún queda por responder el interrogante de la utilidad clínica de este biomarcador para orientar la dosificación del diurético intravenoso en un algoritmo como el propuesto por la Sociedad Europea de Cardiología en sus guías y consensos ^11^^,^^12^. Para esto, deben continuar los esfuerzos por demostrar la eficacia y la seguridad del uso de este algoritmo, como lo pretende hacer un ensayo clínico que se encuentra en curso (NCT04606927) y que intenta responder dicha pregunta de investigación.

Este estudio tiene algunas limitaciones: no se logró recolectar el suficiente número de pacientes para llevar a cabo un análisis multivariado, de forma que las asociaciones descritas pueden estar sujetas a algún sesgo de confusión y se requiere una muestra más grande para evaluar de manera independiente el efecto del sodio urinario en los desenlaces. La poca población incluida se debió a las características del estudio, que dependía de la solicitud del sodio en orina por parte del médico tratante. A su vez, esto demuestra que los niveles de sodio en orina tienen poca acogida en la comunidad médica a pesar de las recomendaciones para su uso, muy posiblemente por cuestiones logísticas y poca familiaridad con la prueba. Por otro lado, no se puede descartar que haya un sesgo de selección en los pacientes a quienes se les practicó el examen de sodio en orina en urgencias.

En conclusión, este estudio sugiere que los pacientes con diagnóstico de falla cardíaca aguda descompensada, con congestión y sodio urinario bajo, tienen mayor riesgo de mortalidad. Los pacientes con sodio urinario inferior a 70 mEq/L tuvieron mayor frecuencia de hospitalizaciones previas, menores niveles de sodio sérico y presión arterial sistémica, y una mayor mortalidad a los 180 días. Se requieren ensayos clínicos o estudios prospectivos que avalen el uso de este biomarcador como guía para la modificación del tratamiento diurético y el establecimiento del mejor punto de corte.
